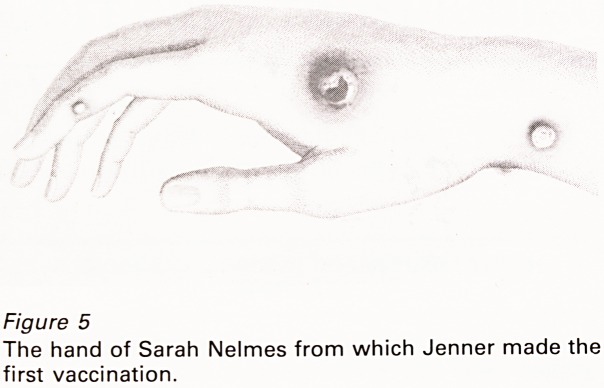# The Natural History of Cowpox

**Published:** 1982

**Authors:** Derrick Baxby

**Affiliations:** Department of Medical Microbiology, Liverpool University, L69 3BX


### The Natural History of Cowpox

### The substance of the Jenner Lecture, read 17th May 1982 in Bristol Royal Infirmary under the auspices of the Jenner Trust

**Derrick Baxby**
Department of Medical Microbiology, Liverpool University, L69 3BX

### INTRODUCTION

Edward Jenner is honoured because between 1796 and 1798 he showed that immunity to smallpox could be induced by inoculation of material from an animal disease which he called 'cowpox'. This paper discusses recent evidence concerning the natural history of cowpox which suggests that earlier views on it may have been incorrect.

In the 19th century valuable information was obtained about cowpox. However these studies in part pre-dated acceptance of the germ theory of disease, and all pre-dated the development of laboratory techniques. Consequently the evidence was based on natural and artificial infection of man and animals. Until the 1930s the terms cowpox, smallpox vaccine, and vaccinia were used synonymously but a breakthrough came in 1939 when A. W. Downie showed that cowpox and smallpox vaccine (i.e. vaccinia) viruses were distinct, although of course immunologically related. Bovine cowpox and vaccinia infections are clinically indistinguishable and laboratory studies are necessary to make a differential diagnosis. Although cases of bovine vaccinia have been reported from Holland, no case has been detected in Britain (Baxby, 1977a). Vaccinia virus is generally regarded as an artificial agent developed for vaccination and its origin and parentage is still the subject of debate (Baxby, 1981).

### TRADITIONAL COWPOX

The view of cowpox which was accepted until recently was based on assumption rather than investigation, although in fairness to earlier workers it must be stressed that they were too busy developing laboratory methods for studying smallpox to pay much attention to an occasional outbreak of cowpox.

The traditional view of cowpox (Table 1) is that it is a bovine infection, enzootic in dairy cattle. Lesions occur on the teats (Figure 1A) and infection is transmitted by milking, to other cows and also to dairy workers for whom it is an occupational hazard. Bovine cowpox is a relatively trivial infection and not notifiable, so that outbreaks are usually only investigated when human cases are reported. Human cowpox, although producing an infection usually more severe than primary vaccination, does not usually require specialist attention.

**Table 1**

**Cowpox – The Traditional View***

| *Case* | *Cattle Infected* | *Farm Worker* | *Age* | *Days in Hospital* |
|---|---|---|---|---|
| Tyson | + | – | – | – |
| '188' | + | – | – | – |
| Dorchester | + | + | A | – |
| Exeter | + | + | A | – |

A = Adult

*Details of cases from Baxby (1977b)

### ENZOOTIC PSEUDOCOWPOX

If in fact cowpox is enzootic in dairy cattle, certain criteria should be met. The validity of these criteria can be assessed by using as a basis for comparison pseudocowpox which has been studied in detail in South-West England. Pseudocowpox (paravaccinia), is caused by a parapoxvirus. The virus produces typical 'ring sores' on the teats of dairy cattle (Figure 1B) and is transmissible to man, producing 'milker's nodes' (Figure 2). Its clinical separation from cowpox infection is not always certain in cattle or man but the correct diagnosis can be established by examining crusts or exudates from the lesion in the electronmicroscope. Pseudocowpox virus is somewhat narrower and has a conspicuous surface structure absent from cowpox (Figure 3). Cowpox and pseudocowpox viruses are placed in different poxvirus genera and show no cross-immunity.

*Figure 1*
Cow's teat showing a lesion caused by (A) cowpox and a 'ring sore' caused by (B) pseudocowpox virus. (Courtesy of the Veterinary Record, from Gibbs et al., 1973)

*Figure 2*
Human infection (milker's nodule) caused by pseudocowpox virus. (Courtesy of Dr. J. Nagington)

*Figure 3*
Electronmicrograph of: (A) cowpox; (B) pseudocowpox viruses. (Negative stain, X100,000)

Pseudocowpox was undoubtedly one of the types of 'spurious cowpox' of which Jenner warned (Baxby, 1981).

1. To be considered enzootic an infection must be present in a sufficiently high proportion of the proposed reservoir species to ensure its continued circulation. If infection is clinically obvious the current incidence should be measurable. Pseudocowpox is widespread in British cattle; Nagington et al. (1966) detected it in $^{14}/_{16}$ herds and Gibbs and Osborne (1974) found it in many herds and in 13% of 358 dairy cattle seen in abattoirs. In both these surveys diagnosis was confirmed by electron microscopy.
2. If the infection produces an adequate serological response, antibody should be detectable in animals and will give an estimate of overall incidence. Pseudocowpox does not produce a very good serological response but other studies have shown that 8% of Dutch pigs have antibody to swinepox (de Boer, 1975) and 19% of cattle in South-West England have antibody to bovine herpes virus (Rweyamamu et al., 1969).
3. If the disease is infectious for man, human cases should be traceable to the animal reservoir. Human infection with pseudocowpox virus is relatively painless and probably only a small proportion is reported. Nevertheless examination of the Public Health Service Weekly Reports shows clearly that pseudocowpox is an occupational hazard for farmworkers, veterinary surgeons and students, and abattoir workers. Virtually all these cases are traceable to infected animals.
4. The geographical distribution of the disease should, within reason, be that of the host species. Pseudocowpox occurs world wide so presumably the virus was exported along with the cattle before quarantine restrictions were introduced.

Clearly pseudocowpox is a good example of a trivial virus infection which is enzootic in cattle.

### COWPOX TODAY

Now, what is the situation with cowpox?

1. The reported incidence of bovine cowpox is very low, probably only 6 or so outbreaks since 1965. This is not simply due to under-reporting of a trivial infection, because the extensive surveys which established the incidence of pseudocowpox detected only 1 outbreak of cowpox (Gibbs et al., 1973). This suggests that clinically obvious bovine cowpox is rare.
2. Cowpox virus induces a good antibody response in naturally infected cattle which persists for at least 2 years (Baxby & Osborne, 1979). If cowpox is enzootic in dairy cattle one might expect perhaps 10% of randomly selected animals to have antibody, and some should have high titres indicating recent recovery. However antibody surveys showed no evidence of active cowpox in 422 randomly obtained serum samples nor in 654 samples from cattle in areas where human cases had occurred. Of the 1076 samples only 7 had any neutralising antibody, and only 1 had a reasonably high titre (Baxby, 1977b). This again suggests an extremely low incidence of bovine cowpox.
3. Human cowpox is rare. Important here is the evidence that human cowpox is a relatively severe infection (Figure 4) and perhaps more severe than earlier accounts suggest. Possibly an occasional farmworker may shrug it off, but medical aid will usually be sought and few cases will be missed; in fact about half the patients are admitted to hospital (Baxby, 1977b). Despite this human cases are reported very rarely and average no more than 1 per year, far fewer than the reported incidence of the much less severe pseudocowpox. Equally important is the fact that the majority of these cases have no contact with cattle, infected or otherwise. Clearly human cowpox cannot be regarded as an occupational hazard, and a perhaps more accurate view of cowpox is shown in Table 2.

The inevitable conclusion to be drawn is that cowpox is not enzootic in cattle. Cows do get infected and may transfer infection to farm-workers in what used to be regarded as the traditional way. However it seems clear that 'cowpox' virus must circulate and be maintained in a species other than the cow and that bovine and some human infections are contracted accidentally from an unknown reservoir.

### COWPOX YESTERDAY

It is unlikely that cowpox ever was enzootic in cattle although it is impossible to be certain; before the development of laboratory methods there would have been confusion between cowpox, and vaccinia, pseudocowpox, and the now-extinct horsepox (Baxby, 1981). Last century both Crookshank (1889) and Seaton (1868) collected data which indicated that cowpox was no more common then than it is now. And of course Jenner had difficulty in obtaining vaccine material; after the initial vaccination of Phipps with

*Figure 4*
Human cowpox. (Courtesy of Mr. J. McNae)

**Table 2**

**Cowpox – A More Realistic View***

| *Case* | *Cattle Infected* | *Farm Worker* | *Age* | *Days in Hospital* |
|---|---|---|---|---|
| Tyson | + | – | – | – |
| Taunton | + | + | A | – |
| Bristol | – | – | 17 | – |
| Penrith | – | – | A | 7 |
| Middles-brough | – | – | 8 | 24 |
| Burnley | – | – | 14 | 21 |

A = Adult

*Details of cases from Baxby (1977b)

*Figure 5*
The hand of Sarah Nelmes from which Jenner made the first vaccination.

virus from the hand of Sarah Nelmes (Figure 5) he had to wait 2 years until he could continue. Later he had to obtain supplies from others, and there is ample evidence that early demands could not always be met from natural sources (Baxby, 1981). Also, if cowpox had been enzootic in cattle it would have been distributed world-wide, as was pseudocowpox, in the days before quarantine restrictions were established. It is perhaps significant that cowpox has not been recorded outside Britain and W. Europe. So it seems likely that cowpox never was enzootic in cattle.

### UNKNOWN RESERVOIR

So far there is no real information about the reservoir species of 'cowpox' virus which is presumed to be a small wild mammal, most probably a rodent. Perhaps the isolated human and bovine cases occur when objects such as barbed wire, brambles etc. become temporarily contaminated. Although the reservoir has not been identified, interesting information has been obtained. Cowpox virus infected 3 cheetahs at Whipsnade Zoo in 1977, killing 2 of them. Attempts were made to trace the source without success (Baxby et al., 1982). In 1978 cowpox virus killed 2 more cheetahs in another zoo and also a domestic cat (Baxby et al., 1979a) and 3 more infected cats have been seen during 1982; in none of these cases was the source traced. It is unlikely that the domestic cat is the reservoir. If so, it would have been recognised earlier, either for itself or through infecting the pets' owners. However it is possible that there is a significant incidence in semi-wild farm cats. These are not petted nor usually treated when sick, and would be in contact with the likely reservoir. Clearly more work is required here.

### EVENTS ABROAD

So far this account has described the situation in Britain but equally interesting events have occurred abroad. Poxvirus infections have killed animals in Moscow Zoo (Marennikova et al., 1977) and a number of outbreaks of pox in elephants has occurred in Germany (Baxby 1977a, Baxby & Ghaboosi 1977). The reservoir of the German virus has not been traced, but that of the Russian virus is a small wild rodent similar to a gerbil. Detailed laboratory studies have shown that cowpox virus and the Russian and German isolates are closely-related but not identical (Baxby et al., 1979b). Fortunately Britain is an island and only cowpox is indigenous here so problems of identification do not arise.

This susceptibility of rare and valuable animals is an unusual and apparently new problem. These animals are endangered species in their natural environment, and are brought or bred here in order to conserve them. Evidently they now face new and serious threats in their new, supposedly safe, habitat.

### CONCLUSIONS

What would Jenner have thought of this? If alive today he would have been particularly impressed by the eradication of smallpox – made possible by the vaccine he pioneered. However he was also interested in natural history and would, I hope, have been intrigued by talk of cats and cheetahs. Certainly despite all the attention paid to it since 1796, there is still a lot to be learned about the natural history of his 'variolae vaccinae'.

### REFERENCES

BAXBY, D. (1977a) Poxvirus hosts and reservoirs. *Arch.Virol.* **55**, 169.

BAXBY, D. (1977b) Is cowpox misnamed? A review of 10 human cases. *Br.Med.J.* **1**, 1379.

BAXBY, D. (1981) *Jenner's Smallpox Vaccine.* Heinemann Educational Books, London.

BAXBY, D. and GHABOOSI, B. G. (1977) Laboratory characteristics of poxviruses isolated from captive elephants in Germany. *J.gen.Virol.* **37**, 407.

BAXBY, D. and OSBORNE, A. D. (1979) Antibody response in natural bovine cowpox. *J.Hyg.* **83**, 425.

BAXBY, D., ASHTON, D. G., JONES, D. et al. (1979a) Cowpox virus infections in unusual hosts. *Vet.Rec.* **109**, 175.

BAXBY, D., ASHTON, D. G., JONES, D. et al. (1982) An outbreak of cowpox in captive cheetahs: virological and epidemiological studies. *J.Hyg.* (in press).

BAXBY, D., SHACKLETON, B. W., WHEELER, J. et al. (1979b) Comparison of cowpox-like viruses isolated from European Zoos. *Arch.Virol.* **61**, 337.

BOER, G. F. de (1975) Swinepox. Virus isolation, experimental infections and the differentiation from vaccinia virus infections. *Arch.Virol.* **49**, 141.

CROOKSHANK, E. M. (1889) *The History and Pathology of Vaccination.* H. K. Lewis, London.

DOWNIE, A. W. (1939a) A study of the lesions produced experimentally by cowpox virus. *J.Path.Bact.* **48**, 361.

DOWNIE, A. W. (1939b) The immunological relationship of the virus of spontaneous cowpox to vaccinia virus. *Brit.J.Exp.Path.* **20**, 158.

GIBBS, E. P. J. and OSBORNE, A. D. (1974) Observations on the epidemiology of pseudocowpox in South-West England and South Wales. *Br.Vet.J.* **130**, 150.

GIBBS, E. P. J., JOHNSON, R. H. and COLLINGS, D. H. (1973) Cowpox in a dairy herd in the United Kingdom. *Vet.Rec.* **92**, 56.

MARENNIKOVA, S. S., MALTSEVA, N. N., KORNEEVA, V. I. et al. (1977) Outbreak of pox disease among carnivora (*Felidae*) and Edentata. *J.Inf.Dis.* **135**, 358.

NAGINGTON, J., TEE, G. H. and SMITH, J. S. (1966) Milker's nodule virus infections in Dorset and their similarity to orf. *Vet.Rec.* **78**, 305.

RWEYAMAMU, M. M., JOHNSON, R. H. and LAURILLARD, R. E. (1969) Serological findings in bovine herpes mammillitis. *Br.Vet.J.* **125**, 317.

SEATON, E. C. (1868) *Handbook of Vaccination.* MacMillan, London.

By the end of 1982, 9 more cases of feline cowpox had been detected, 6 in domestic cats, and one more cheetah (Baxby and Gaskell, unpublished data)

## Figures and Tables

**Figure 1 f1:**
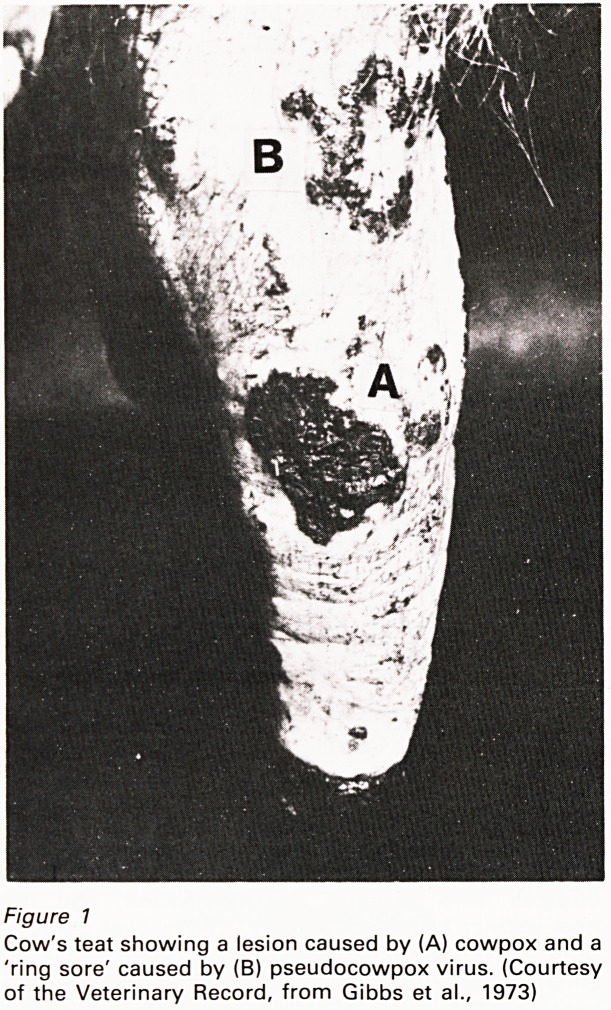


**Figure 2 f2:**
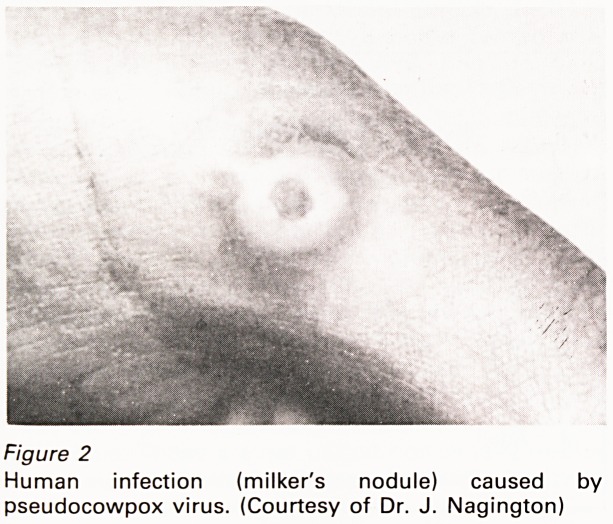


**Figure 3 f3:**
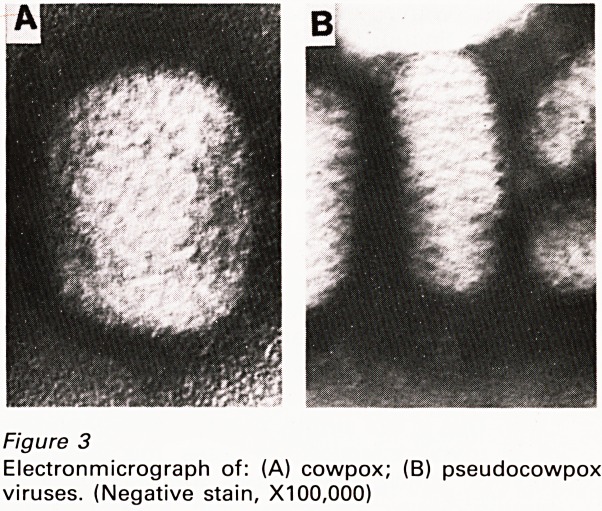


**Figure 4 f4:**
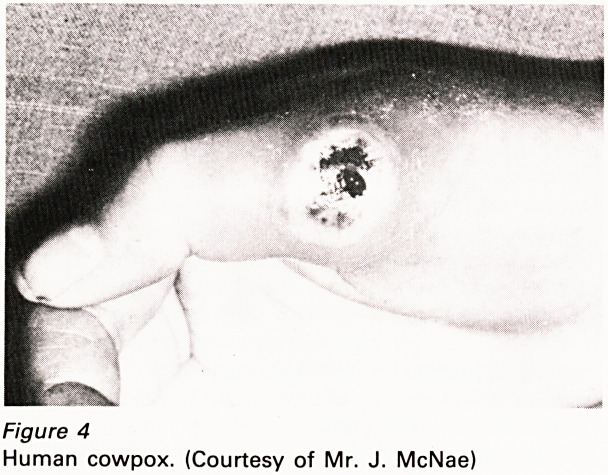


**Figure 5 f5:**